# Short-term and long-term effects of the COVID-19 pandemic on child psychological well-being: a four-wave longitudinal study

**DOI:** 10.1007/s00787-023-02215-7

**Published:** 2023-04-29

**Authors:** Samuel Essler, Natalie Christner, Markus Paulus

**Affiliations:** 1 Developmental Psychology, Ludwig-Maximilians-Universität München, Leopoldstr. 13, 80802 Munich, Germany; 2 FOM University of Applied Sciences, Essen, Germany

**Keywords:** COVID-19, Natural experiment, Parent–child relationship quality, Parental stress, Child well-being, Child problem behavior

## Abstract

As the COVID-19 pandemic further unfolds, it becomes a key theoretical and practical question to identify trajectories of child psychological well-being and to explore risk and resilience factors for developmental adjustment. The current study addressed this research gap by means of an ecological design: A (lockdown)–B (relaxation)–B (relaxation)–A (lockdown). We collected parental reports via online questionnaires over four measurement occasions during the COVID-19 pandemic in Germany (non-probabilistic sample): from the first lockdown (T1—spring 2020, *N* = 1769) to the following period of relaxation (T2—summer 2020, *n* = 873; T3—fall 2020, *n* = 729) on to the second lockdown (T4—winter 2020/21, *n* = 748). Key measures at T1–T4 were child emotional and behavioral problems as well as hyperactivity, child emotional and family-related well-being, parental strain, and parent–child relationship quality. We found evidence for quadratic growth models. While child problem behaviors (*b* = 0.32, *p* < 0.001) and emotional well-being (*b* = − 0.33, *p* < 0.001) improved after the first lockdown during subsequent periods of relaxation before worsening again in the second lockdown, child family-related well-being steadily decreased over all four measurement points (T1–T2: *p* < 0.001; T2–T3: *p* = 0.045; T3–T4: *p* = 0.030). Importantly, parental stress emerged as a strong risk factor (*p*s < 0.11) and the parent–child relationship quality constituted a resilience factor (*p* = 0.049) for child psychological well-being. These findings have major implications for policies aiming to further child health during the COVID-19 pandemic.

**Supplementary Information:**

The online version contains supplementary material available at 10.1007/s00787-023-02215-7.

## Introduction

The COVID-19 pandemic constitutes one of the major large-scale sociohistorical disruption of the twenty-first century. In the first year of the pandemic, strict lockdowns and periods of loosened restrictions followed each other and presented major challenges for child well-being and psychosocial adjustment (e.g., [9, 10, 58]. While increasing (longitudinal) literature reports on child mental health during the COVID-19 pandemic (e.g., [6, 11, 19, 25, 27, 46, 62, 68], we know little about the differences between long-term effects (i.e., of the pandemic generally independently of lockdowns) and short-term effects (i.e., specific effects of lockdowns). This requires longitudinal work spanning multiple alternations of lockdowns and relaxations. As opposite to lockdowns, relaxations thereby refer to time periods of less restrictive public health measures such as less social distancing, reopening of educational facilities, and less home-office obligations in many countries [21, 34]. The present study aimed at adding a novel perspective on this issue of great societal concern by investigating trajectories of child mental health and psychosocial adjustment during the COVID-19 pandemic in Germany relying on parental report.

### Trajectories and determinants of child psychological well-being

Major developmental theories, such as life course theory [5, 17] and ecological systems theory [8], propose that macrostructural disruptions impact child well-being and development. While some aspects of crisis might directly impact developmental trajectories (e.g., restrictions on interactions with peers during lockdowns), others might affect children indirectly through their caregivers (e.g., home-office obligations leading to increased parental stress). Within these theoretical frameworks, the COVID-19 pandemic can be conceptualized as an unexpected sociohistorical change deflecting trajectories of child well-being. However, the particular shape of developmental deflections in the context of the COVID-19 pandemic remains to be specified.

From a theoretical perspective, there are two child developmental trajectories that seem especially likely during the COVID-19 pandemic. First, following the Parenting Stress Model [1, 30], environmental disruptions during COVID-19, such as home confinement, restrictions on social interactions, and changed working conditions, should increase caregivers’ strain and subsequently negatively impact child adjustment. As environmental stressors and COVID-19-related uncertainty are most intense during lockdowns, one would expect caregivers’ stress to culminate during this time period limiting parental capacity to address children’s stress and anxiety. Consequently, children’s well-being should be especially compromised during lockdowns. A host of studies provides empirical support for notions of lockdowns as particularly challenging phases for child and family functioning [10, 11, 19, 25, 46, 56, 68, 70, 75]. Thus, one would expect children’s well-being to be especially compromised during lockdowns and recover during relaxations (short-term effects resulting in a wave-like trajectory).

Second, child developmental trajectories might follow a linear trend with consistently decreasing well-being and increasing problem behavior during the COVID-19 pandemic (long-term effects of the pandemic in general independently of specific lockdowns resulting in a linear trajectory). Following ecological systems theory [8], many COVID-19-related stressors might continue to constitute substantial proximal threats to child well-being even during times of loosened restrictions. Among those are socioeconomic stressors, such as caregiver job loss or reduction of working hours, financial insecurity, continuing changes of educational caregiving and schooling (e.g., combination of offline and online learning), as well as psychological stressors such as expectations and cognitions concerning the further course of the pandemic or anxiety concerning a COVID-19 infection of oneself or of close others (e.g., [37]; 43; 53). From a developmental perspective, this would suggest that the COVID-19 pandemic despite its wave-like loosening and tightening of public health measures poses a continual risk factor for child adjustment [12, 14, 15, 36, 40, 42, 46, 51, 70, 74, 79]. In addition, while governmental responses to the COVID-19 pandemic were direct and swift during the first months and highly supported by the public, political conflict and frustration in the public increased as the pandemic continued [7, 37]. This increasing uncertainty and frustration could mirror itself in children’s decreasing well-being.

Previous longitudinal work on the effects of the COVID-19 pandemic (for meta-analyses, see [45, 60]) demonstrated reduced psychological well-being from pre-pandemic to lockdown [77] with recoveries from lockdown to subsequent relaxations [6, 19, 66] but reductions in child well-being from the first to subsequent lockdowns [7, 27, 62–64]. Other work reports positive effects of children’ and youth’s pandemic-related stressors during the first lockdown on externalizing and internalizing symptoms later in the pandemic [67]. Thus, longitudinal evidence concerning trajectories of child psychological well-being and mental health remains inconclusive. The current study addresses this research gap by testing two theoretical trajectories (wave-like trajectory and linear trajectory) against each other and thereby contributing novel insights on how children cope with the unfolding COVID-19 pandemic.

Theoretical models on child mental health and well-being suggest important interindividual differences in child adjustment to the pandemic [58]. That is, it is predicted that some children will be able to cope better with the current challenges and show less adjustment difficulties than others. For example, parent–child relationship, pre-existing family vulnerabilities, as well as beliefs and communication within the family are proposed to constitute potential resilience factors. Adapting the propositions of the Family Stress Model [3, 44, 47] to the context of the pandemic, COVID-19-induced social disruptions could lead to elevated caregiver stress, which in turn could favor inadequate parenting practices (e.g., harsh, authoritarian parenting) resulting in interindividual differences in child adjustment problems. Indeed, studies report associations between COVID-19-related parental distress, daily parenting, and parental attachment style and child externalizing symptoms and adjustment [19, 22, 35, 41, 54, 66]. However, there is no work assessing the role of parental distress as risk factor for broader trajectories of child well-being leaving open the question if and to what extent parental distress compromises child mental health during different phases of the COVID-19 pandemic.

Theoretical frameworks of resilience have claimed that close social relationships are among the most important resilience factors for child well-being during social disruptions [13, 48–50, 69, 71]. In the context of the COVID-19 pandemic, the parent–child relationship quality could therefore function as a resilience factor in children’s developmental trajectories as also suggested by the previous work [4, 16, 55]. However, there is little evidence on the long-term effects of the parent–child relationship quality on child well-being trajectories during the pandemic. One notable exception is work by Ravens-Sieberer et al. [62-64] who demonstrated that family/parental support has short- and long-term effects on child well-being. The present study directly investigates the theoretical possibility of the parent–child relationship functioning as important resilience factor.

### The current study

The research question of the present study was twofold. First, we aimed to examine the shape of developmental trajectories (wave-like or linear) of parental strain and child problem behavior and well-being over the first year of the COVID-19 pandemic in Germany (i.e., across four measurement points). Second, we investigated whether parental strain would act as a risk factor and parent–child relationship quality as a resilience factor for children’s well-being and behavior by aggravating or mitigating negative effects of the COVID-19 pandemic. Importantly, the present study is among the first to rely on an A (lockdown)–B (relaxation)–B (relaxation)–A (lockdown) design constituting a naturalistic ecological approach to more systematically investigate effects of lockdowns on child mental health. While findings from previous studies comparing pre-pandemic to lockdown [77] or lockdown to further relaxations [6, 19, 66] or lockdowns [7, 27, 62–64] provide important first evidence on lockdown effects, the present ecological design with different conditions takes an important first step to differentiate short-term effects from long-term effects of the pandemic.

Thus, the current study offers a novel methodological perspective on understanding trajectories of child psychological well-being during the COVID-19 pandemic in two ways: (1) almost all previous longitudinal studies employed a pre-pandemic–A design [77], an A–B design [6, 19, 66], or an A–A–B design [7, 27, 62–64]. Our study is among the first to employ an ecological design (A–B–B–A) with two changes between lockdown and relaxation; (2) previous longitudinal research (see above) does not use two relaxation periods to control for confounding factors that could impact improvements from lockdown to relaxation (e.g., seasonality). Taken together, the present study presents a unique opportunity to differentiate short-term from long-term effects and at the same time control for confounding variables to a greater degree than previous work.

Based on the above theoretical considerations, we hypothesized that (1) child problem behavior, child emotional well-being, and parental strain would follow a wave-like trajectory (i.e., recovering during relaxation periods). In addition, we hypothesized that (2) child family-related well-being would steadily decline as COVID-19-related family stressor remained high during periods of loosened restrictions (i.e., not recovering during relaxation periods). Finally, we hypothesized that (3) parental stress would negatively impact child well-being trajectories by increasing their volatility, while the parent–child relationship quality would positively impact well-being trajectories by decreasing their volatility.

## Methods

### Participants

Participants were primary caregivers of 3 to 10 years old reporting on their own and their children’s well-being. The final sample sizes of the four measurement points (T1–T4) were as follows: *N*(T1) = 1769; *n*(T2) = 873; *n*(T3) = 729; *n*(T4) = 748. Out of the 1769 participants, 361 completed all four measurement points. We excluded additional participants for not consenting to be contacted for follow-up questionnaires (T1: *n* = 1008), for starting the questionnaire but not answering any questions concerning the key study variables (parental strain, child well-being and problem behavior, parental self-efficacy, relationship quality; T1: *n* = 183, T2: *n* = 99, T3: *n* = 117, and T4: *n* = 121), for providing unidentifiable or non-matching ID-codes (T1: *n* = 42, T2: *n* = 157, T3: *n* = 131, and T4: *n* = 160), and for inconsistent or invalid age or gender reports (T1: *n* = 202, T2: *n* = 85, T3: *n* = 67, and T4: *n* = 66). We recruited participants ad hoc in a non-probabilistic fashion via online postings on the lab website and social media, via the lab database containing families affiliated with the lab through earlier contacts, and by words of mouth (i.e., personal invitations through lab members).

We collected data for T1 (end of April to beginning of May 2020) and T4 (end of January to beginning of March 2021) during the first two major lockdowns in Germany. These lockdowns arguably represent the most demanding phases of the COVID-19 pandemic for children and families so far (home-office obligations, closure of educational facilities, and restrictions on social interactions). In contrast, we collected data for T2 (middle of July 2020) during a time when all the major lockdown restrictions had been loosened. Collection of data for T3 (end of October to beginning of December 2020) took place during the case acceleration phase prior to the second lockdown. The present study reports an overall picture with two smaller reports on T1 and T2 having been published previously (references blinded for review).

Table 1 displays the demographic characteristics of the sample. Comparing sociodemographic characteristics from T1 to T4 shows that there are no systematic dropouts (maximum change of 3% points). The majority of the sample was of Western European ethnicity. The current study was approved by the local ethics committee. Participants gave their informed consent.

**Table 1:** Key demographic characteristics of the sample at T1 (*N* = 1769), T2 (*N* = 873), T3 (*N* = 729), and T4 (*N* = 748)

Demographic variable	T1	T2	T3	T4
Vocational degree				
University degree	52%	54%	56%	53%
Vocational training	21%	19%	20%	19%
University of applied sciences degree	15%	16%	15%	17%
Professional academy	8%	8%	7%	8%
Master training	3%	2%	2%	3%
No vocational degree	1%	< 1%	< 1%	< 1%
Current job status				
Home office	43%	31%	29%	44%
Job outside of the home	18%	35%	40%	29%
Parental leave	19%	18%	16%	11%
Reduced working hours	6%	4%	3%	3%
No job	5%	5%	5%	5%
Exempted	4%	2%	1%	2%
Other	6%	5%	6%	5%
Change in attendance of educational institutions as compared to T1				
Yes, my child visits preschool again	–	53%	40%	16%
Yes, my child visits school again	–	34%	45%	17%
No, my child continues to visit an institution	–	6%	14%	36%
Yes, my child visits a daycare center again	–	4%	0%	0%
No, my child continues to visit no institution due to COVID-19	–	3%	0%	28%
No, my child continues to visit no institution	–	1%	0%	3%
Change in further extra familiar childcare (grandparents, nanny, …) as compared to T1				
No, my child continues to receive no extra familial childcare	–	40%	42%	45%
Yes, my child receives extra familial childcare again	–	34%	32%	17%
No, my child continues to receive no extra familial childcare due to COVID-19	–	18%	18%	24%
No, my child continues to receive extra familial childcare	–	8%	8%	15%
Age of child at T1				
3 years	17%	–	–	–
4 years	18%	–	–	–
5 years	17%	–	–	–
6 years	17%	–	–	–
7 years	12%	–	–	–
8 years	9%	–	–	–
9 years	6%	–	–	–
10 years	5%	–	–	–
Gender of child				
Male	52%	50%	50%	49%
Female	48%	50%	50%	51%
Diverse or not specified	< 1%	–	–	–
Gender of primary caregiver				
Male	7%	7%	7%	6%
Female	93%	93%	93%	94%
Diverse or not specified	< 1%	–	< 1%	< 1%
Federal state of residence				
Bavaria	71%	73%	73%	75%
Baden-Württemberg	6%	6%	6%	6%
Berlin	3%	3%	3%	3%
All other 13 other states	< 3%	< 3%	< 3%	< 3%

### Power analysis

We conducted an a-priori statistical power analysis for SEM models with the R-package semPower [52]. By entering the effect size as RMSEA = 0.05, alpha = 0.05, power = 0.80, and 10 degrees of freedom, we obtained a required sample size of 651. Therefore, our objective was a sample of at least *N* = 700 at each measurement point.

### Design and materials

We assessed child emotional and behavioral problems as well as hyperactivity (child problem behavior; based on [26], child emotional and family-related well-being [61], parental strain, and parent–child relationship quality [24]. Specifically, we relied on an ecological A (lockdown)–B (relaxation)–B (relaxation)–A (lockdown) design as methodological opportunity to separate short-term from long-term effects on changes in child well-being. The rationale behind the two relaxation phases as measurement points was to rule out possible confounding factors explaining the changes in well-being from T1 to T2 (e.g., seasonality). At each measurement point, questionnaires comprised three blocks (see below). We asked at each timepoint that the primary caregiver of the target child in terms of time answers the survey.

#### Block 1: demographics and parental strain

##### Demographics (T1–T4)

Demographic questions related to the parents the target child. Concerning the parents, questions addressed the participants’ age (T1–T4), gender (T1–T4), family and partner status (T1), educational degree of self and partner (T1), current job status of self and partner (T1–T4), and housing situation and number of children in the household (T1). In addition, participants indicated to what extent the hours spent taking care of their child increased as compared to before the pandemic (T1–T4). Concerning the target child, questions addressed age (T1–T4), gender (T1–T4), and the changing status of educational institution attendance and other extrafamilial care arrangements (T1–T4).

##### Parental strain (T1–T4)

A scale of three questions assessed parental strain as compared to before the pandemic. In the instructions, participants were asked to consider the specific current phase of the pandemic (i.e., lockdown or relaxation) by focusing on the weeks prior to the present day. Specifically, at T1, participants were asked to answer with respect to the time since the beginning of the curfew restrictions and at T2–T4 with respect to the last three weeks. The items were: “I feel more strained in the current situation than normally”, “The current situation is more challenging for me than normally”, “I feel more stressed out in the current situation than normally”; Cronbach’s *α* (T1) = 0.90, Cronbach’s *α* (T2) = 0.94, Cronbach’s *α* (T3) = 0.93, and Cronbach’s *α* (T4) = 0.93. Participants responded on a Likert scale ranging from 1 (“do not agree at all”) to 5 (“totally agree”). Means across the three items were calculated to form the *parental strain* variable for subsequent analyses.

#### Block 2: child’s situation during the COVID-19 pandemic

##### Child well-being: KIDSCREEN (T1–T4)

We assessed the effects of the COVID-19 pandemic on the child by modifying 12 items from the German translation of the KIDSCREEN-52 Health-Related Quality of Life Questionnaire for Children and Adolescents [61]. It shows acceptable Cronbach’s Alphas for the dimensions (0.76–0.89) and demonstrates good convergent and discriminant validity [61]. The rationale for using items from the KIDSCREEN-52 and not from shorter versions of the KIDSCREEN was that we theoretically distinguished the subscales we chose (see below) as assessing different aspects of well-being and did not want to combine them a priori as in shorter KIDSCREEN versions.

We selected the set of items as described below for three reasons. First, some subscales were inapplicable due to COVID-19-related lockdown restrictions. That is, due to reduced social interactions with friends and decreased attendance of educational institutions, we dropped items relating to these domains. Second, we dropped items with very similar wordings due to time constraints (e.g., we used “was in a good mood” but not “was happy). Third, we chose subscales based on their theoretical relevance for the research questions (e.g., “feelings” but not “physical activities”). Further, we modified the items to address positive and negative changes in quality of life by comparing quality of life at the measurement points to the quality of life before the COVID-19 pandemic. Specifically, parents reported how much more or how much less their child had positive emotions, moods, time for itself and with its parents at the four measurement points as compared to before the onset of the COVID-19 pandemic. Participants answered on a scale from 1 (“clearly less”) to 7 (“clearly more”) with the middle category 4 denoting “no difference”.

The items were (“Compared to the situation before the COVID-19 pandemic, my child (item 1–12) in the last weeks?”): (1) enjoyed life, (2) was in a good mood, (3) had fun (1–3 aggregated to subscale “emotions”; Cronbach’s *α* (T1) = 0.88, Cronbach’s *α* (T2) = 0.91, Cronbach’s *α* (T3) = 0.88, Cronbach’s *α* (T4) = 0.87), (4) was sad, (5) felt so bad that s/he did not want to do anything, (6) was lonely (4–6 aggregated to subscale “moods”; Cronbach’s *α* (T1) = 0.78, Cronbach’s *α* (T2) = 0.83, Cronbach’s *α* (T3) = 0.77, Cronbach’s *α* (T4) = 0.80), (7) was content (single item subscale for “life satisfaction”), (8) had time for himself/herself, (9) was able to do things s/he wanted to do in its free time (8–9 aggregated to subscale “free time”; Cronbach’s *α* (T1) = 0.43, Cronbach’s *α* (T2) = 0.29, Cronbach’s *α* (T3) = 0.16, Cronbach’s *α* (T4) = 0.30—subscale dropped due to low reliability values), (10) felt that its parents had time for it, (11) felt fairly treated by its parents, and (12) has been able to talk to its parents when s/he wanted (10–12 aggregated to subscale “family”; Cronbach’s *α* (T1) = 0.69, Cronbach’s *α* (T2) = 0.73, Cronbach’s *α* (T3) = 0.62, Cronbach’s *α* (T4) = 0.65). We calculated means for the subscales. Given the high intercorrelations between the three subscales relating to children’s emotional well-being (emotions, moods, life satisfaction; *r*s > 0.59 for T1–T4), we computed means across the subscales to yield the variable *emotional well-being* for the subsequent analyses. The subscale “family” was included as *family-related well-being* in the subsequent analyses.

##### Child problem behaviors (T1–T4)

We used three modified subscales (emotional symptoms, conduct problems, and hyperactivity-inattention) of the Strengths and Difficulties Questionnaire (SDQ; [26] based on theoretical considerations to investigate children’s problem behavior. Reliabilities of the SDQ subscales have been reported as acceptable with Cronbach’s *α* = 0.58–0.76 [73]. We asked participants to answer the questions with respect to the last 3 weeks on a 3-point scale (0—“not true, 1—“somewhat true”, and 2—“certainly true”). To make the items compatible with COVID-19-related lockdown restrictions, we adapted and shortened the respective items (e.g., removing references to behavior at school or toward other children). To avoid ambiguous item phrasing (e.g., “Often unhappy, depressed, or tearful”) and to keep the structure of items comparable, we also modified the other items as follows: emotional problems (“Often complains of headaches”, “Has many worries”, “Often unhappy”, “Nervous or clingy”, “Has many fears”; Cronbach’s *α* (T1) = 0.77, Cronbach’s *α* (T2) = 0.69, Cronbach’s *α* (T3) = 0.68, Cronbach’s *α* (T4) = 0.70), conduct problems (“Often has temper tantrums”, “Generally obedient”, “Often fights”, “Often lies or cheats”, “Steals from home”; Cronbach’s *α* (T1) = 0.70, Cronbach’s *α* (T2) = 0.65, Cronbach’s *α* (T3) = 0.60, Cronbach’s *α* (T4) = 0.63), and hyperactivity (“Restless, overactive”, “Constantly fidgeting”, “Easily distracted”, “Reflects”, “Sees tasks through to the end”; Cronbach’s *α* (T1) = 0.66, Cronbach’s *α* (T2) = 0.66, Cronbach’s *α* (T3) = 0.66, Cronbach’s *α* (T4) = 0.65). We computed sum scores for the subscales. Due to their substantial intercorrelations (*r*s > 0.28 for T1–T4), we calculated means across the three subscales to yield an overall *problem behavior* variable for subsequent analyses.

#### Block 3: parent–child relationship quality (T1–T3)

To assess the quality of the parent–child relationship, we modified eight items from the Network of Relationships Inventory while keeping the original subscales (NRI; [24]. Previous work demonstrated its acceptable reliability values with Cronbach’s *α* > 0.60 [24]. Participants responded on a scale ranging from 1 (“never”) to 5 (“very often”). Combining the items into four pairs resulted in the subscales “Intimacy” [Cronbach’s *α* (T1) = 0.88, Cronbach’s *α* (T2) = 0.89, Cronbach’s *α* (T3) = 0.90; example item: “My child tells me what he/she is thinking”], “Admiration” [Cronbach’s *α* (T1) = 0.68, Cronbach’s *α* (T2) = 0.71, Cronbach’s *α* (T3) = 0.72], “Conflict” [Cronbach’s *α* (T1) = 0.80, Cronbach’s *α* (T2) = 0.84, Cronbach’s *α* (T3) = 0.86], and “Dominance” [Cronbach’s *α* (T1) = 0.67, Cronbach’s *α* (T2) = 0.72, Cronbach’s *α* (T3) = 0.74]. As the positive aspects (intimacy and admiration; *r*s > 0.31 for T1–T3) and the negative aspects (conflict and dominance; *r*s > 0.23 for T1–T3) correlated most strongly with each other, we computed means across the positive/negative aspects to yield the variables *positive/negative relationship quality* for subsequent analyses. Parenting self-efficacy was assessed at T1 but is not relevant for the current analyses.

### Procedure

The questionnaires of all measurement points were hosted on Qualtrics with approximately 10–15 min completion time. At the beginning of each questionnaire, instructions informed participants about the purpose of the study and data privacy. Subsequently, participants completed the three blocks of the questionnaire.

### Analyses

We ran all analyses in R, version 4.1.2 [59]. In the first part of the analyses, we aimed to describe the trajectories of the key variables (parental strain, child emotional well-being, child family-related well-being, and child problem behavior) across the four measurement points. To that end, we fitted Latent Growth Models (LGM) in a Structural Equation Modeling framework. For estimating linear growth, we fitted LGMs with intercepts and linear slopes only. For estimating quadratic growth, we fitted LGMs with intercepts, linear slopes, and quadratic slopes. In the second part of the analyses, we aimed to predict changes in child outcome variables between T1 and T2 and from T3 to T4. To that end, we fitted True Intraindividual Change (TIC) Models. TIC models constitute a statistical approach to test predictors of intraindividual change between two measurement points [72]. To that end, variables are modeled as baseline and change variables. Latent baseline variables represent the first measurement of the respective variable (T1) and latent change variables represent change over time in comparison to the precedent measurement point. Our procedure followed previous research [57]. For example, to model change between T1 and T2 in child well-being, a latent baseline variable predicted all measurements (T1–T4) of child well-being and a latent change variable predicted child well-being at T2, T3, and T4. To increase power and to avoid bias due to missing data, we imputed missing data via predictive mean matching using the mice package, version 3.14.0 [18, 76]. We imputed all missing data of the key variables of all measurement points. That resulted in imputation of at least one variable of 912 participants at T1, of 1058 participants at T3, and of 1027 participants at T4. We used the package semTools, version 0.5.5 [33] to estimate the respective models on the imputed dataset (growth models for the trajectories with the function growth.mi() and TIC models with sem.mi() using a maximum-likelihood estimator). Data supporting our analyses are openly available on OSF at https://osf.io/5zrm2/.

## Results

Descriptives of the key variables for all measurement points are presented in Table 2.

**Table 2:** Means (M) and standard deviations (SD) for the key variables at all measurement points

	T1		T2		T3		T4	
Variable	M	SD	M	SD	M	SD	M	SD
Parental strain	3.97	1.03	3.23	1.18	3.22	1.17	4.09	0.96
Child emotional well-being (Kidscreen)	3.42	1.16	4.29	1.12	3.88	0.78	3.21	0.88
Child family-rel. well-being (Kidscreen)	4.25	1.10	4.04	0.85	3.96	0.65	3.84	0.94
Child problem behavior	3.44	1.85	2.86	1.64	2.69	1.64	3.42	1.82
Relationship quality (positive) (NRI)	4.30	0.53	4.14	0.52	4.16	0.51	–	–
Relationships quality (negative) (NRI)	2.73	0.50	2.80	0.51	2.77	0.52	–	–

For all variables, higher scores reflect a stronger expression of the respective variable (e.g., more parental strain, more child well-being, …)

### Trajectories of key variables

**Fig. 1:**
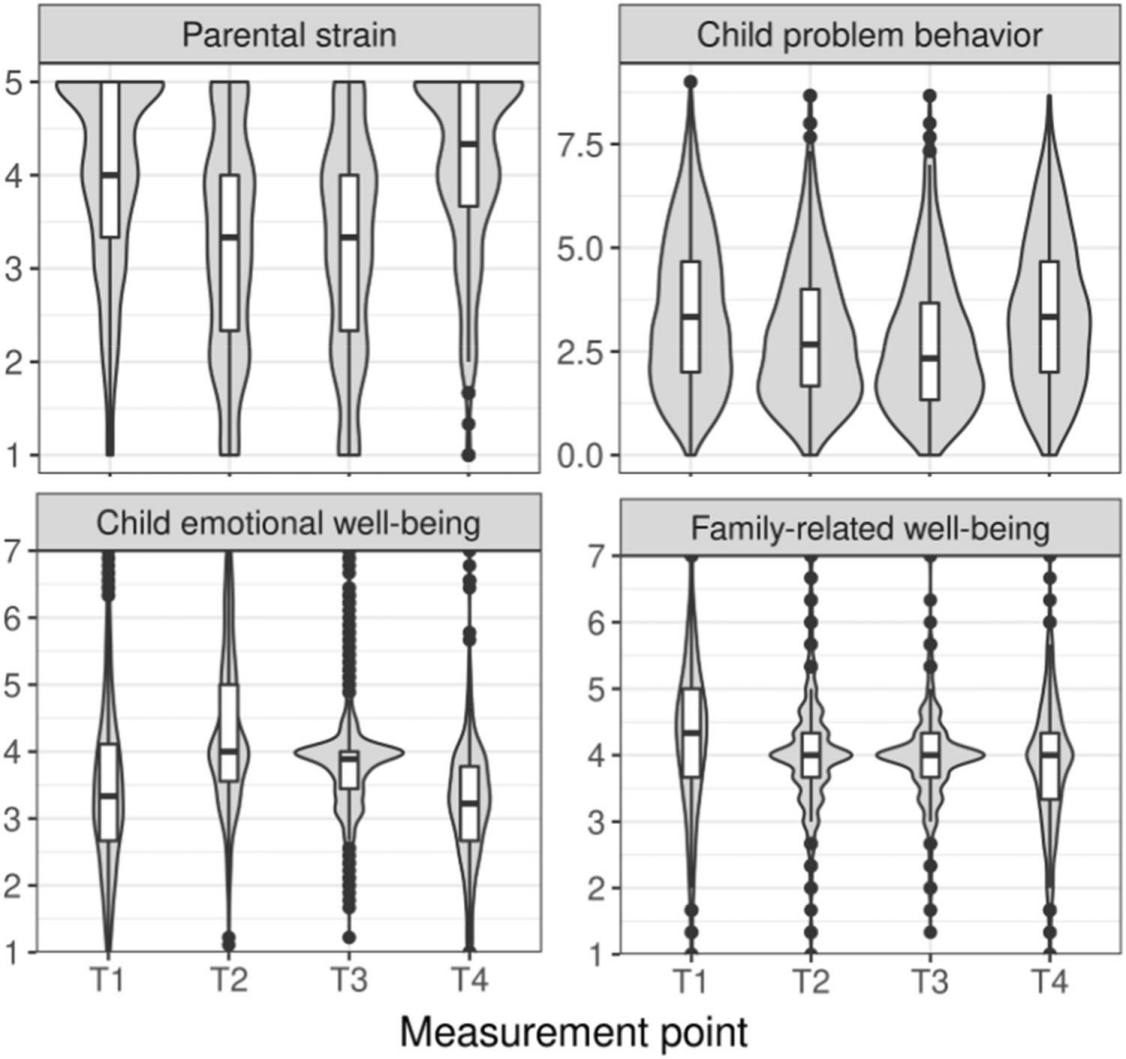
Trajectories of parental strain, child problem behaviors, child emotional well-being, and child family-related well-being across the four measurement points. Violin plots depict the distribution and boxplot of each variable at each measurement point. Potential range of the variables: parental strain 1–5, child problem behavior 0–10, child emotional well-being 1–7, and family-related well-being 1–7

Figure 1 depicts means of parental strain, child problem behavior, child emotional well-being and child family-related well-being across time. We examined trajectories of these variables by fitting linear growth models (with intercepts and linear slopes) and quadratic growth models (with intercepts, linear and quadratic slopes). For the trajectory of *parental strain*, the quadratic growth model had an excellent fit with *χ*^2^ (1, *n* = 1769) = 0.41, *p* = 0.522, RMSEA = 0.000, SRMR = 0.005, CFI = 1.00. Intercepts of the intercept factor (*b* = 3.98, *p* < 0.001), linear slope factor (*b* = − 1.17, *p* < 0.001), and quadratic slope factor (*b* = 0.40, *p* < 0.001) were significant. The significant quadratic slope factor means that, on average, parental strain follows quadratic growth across measurement points. Likewise, variances of the intercept factor (*b* = 0.54, *p* = 0.001), linear slope factor (*b* = 0.56, *p* = 0.013), and quadratic slope factor (*b* = 0.06, *p* = 0.003) were significant, speaking for interindividual differences in the rate of change. In comparison, fitting a linear growth model resulted in negative estimated variances and thus in no interpretable model.

For the trajectory of *child problem behavior*, the quadratic growth model had an excellent fit with χ^2^ (1, *n* = 1769) = 2.64, *p* = 0.104, RMSEA = 0.030, SRMR = 0.015, CFI = 0.998. Both intercepts and variances of the intercept factor (intercept: *b* = 3.46, *p* < 0.001, variance: *b* = 2.69, *p* < 0.001), linear slope factor (intercept: *b* = − 0.97, *p* < 0.001, variance: *b* = 1.10, *p* = 0.012), and quadratic slope factor (intercept: *b* = 0.32, *p* < 0.001, variance: *b* = 0.11, *p* = 0.001) were significant. These results show quadratic growth of child problem behavior across measurement points and interindividual differences in the rate of change. Fitting a linear growth model resulted in negative estimated variances and thus in no interpretable model.

For the trajectory of *child emotional well-being*, the quadratic growth model had an acceptable fit with *χ*^2^ (1, *n* = 1769) = 56.99, *p* < 0.001, RMSEA = 0.178, SRMR = 0.069, CFI = 0.790. Both intercepts and variances of the intercept factor (intercept: *b* = 3.49, *p* < 0.001, variance: *b* = 0.32, *p* = 0.049), linear slope factor (intercept: *b* = 0.90, *p* < 0.001, variance: *b* = 0.44, *p* = 0.045), and quadratic slope factor (intercept: *b* = − 0.33, *p* < 0.001, variance: *b* = 0.06, *p* = 0.010) were significant. These findings speak for quadratic growth across measurement points and interindividual differences in the rate of change. Fitting a linear growth model revealed no interpretable model.

For the trajectory of *child family-related well-being*, the quadratic growth model had a good fit with *χ*^2^ (1, *n* = 1769) = 1.83, *p* = 0.176, RMSEA = 0.022, SRMR = 0.012, CFI = 0.996. Intercepts of the intercept factor (*b* = 4.24, *p* < 0.001) and the linear slope factor (*b* = -0.19, *p* = 0.004) were significant. The intercept of the quadratic slope factor was not significant (*b* = 0.02, *p* = 0.225), suggesting that the average intraindividual change is not described by a quadratic parameter. The variance of the intercept factor (*b* = 0.46, *p* = 0.007), but not of the linear slope factor (*b* = 0.31, *p* = 0.126) and quadratic slope factor (*b* = 0.03, *p* = 0.095) were significant, speaking for no interindividual differences in the rate of change in a quadratic model. Fitting a linear growth model resulted in negative estimated variances and thus no interpretable model. Since the descriptive pattern of means across times clearly suggested a linear trajectory for family-related well-being, we additionally computed dependent sample t test on the non-imputed data to compare family-related well-being between measurement points. Family-related well-being decreased significantly from T1 to T2, *t*(859) = 4.27,* p* < 0.001, from T2 to T3, *t*(494) = 2.01, *p* = 0.045, and from T3 to T4, *t*(474) = 2.17, *p* = 0.030. This pattern suggests on average linear decrease in family-related well-being.

### True intraindividual change models

**Fig. 2:**
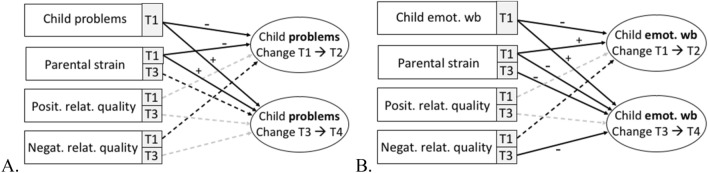
Results of the true intraindividual change models, predicting individual change in child problem behavior (**A**) and child emotional well-being (**B**) from T1 to T2 and from T3 to T4. Signs (±) indicate the sign of the parameter estimates of significant paths. Solid arrows: *p* < 0.05; dashed arrows (black): *p* < 0.10; dashed arrows (grey): *p* > 0.10

To predict changes between measurement points, we computed one TIC model for each child variable (problem behavior, emotional well-being, and family-related well-being). As outcome variables, we predicted the change from T1 to T2 and from T3 to T4 of the respective child variable within the same model. As predictors for the change from T1 to T2, we considered the respective child variable at T1 as well as parental strain, positive relationship quality, and negative relationship quality at T1. As predictors for the change from T3 to T4, we considered the respective child variable at T1 and parental strain at T1 (as control variable) as well as parental strain, positive relationship quality, and negative relationship quality at T3. We specified the covariance between parental strain, positive relationship quality, and negative relationship quality measured at T1 and its respective measurement at T3 to be estimated to account for stability. Covariance between parental strain at T1 and the respective child variable at T1 was additionally estimated. When reporting effect sizes, we rely on the benchmarks for standardized parameter estimates as presented in Acock [2] with *β* < 0.2 being considered as a weak, 0.2 < *β* < 0.5 a moderate, and *β* < 0.5 a strong effect. Parameter estimates for all models are presented in Table 3. An overview of the results is depicted in Fig. 2.

**Table 3:** Means (M) and standard deviations (SD) for the key variables at all measurement points

	T1→T2			T3→T4		
Variable	*β*	SE	*p*	*β*	SE	*p*
Child problem behaviors						
Child problem behav. T1	− 0.52	0.12	< 0.001	0.09	0.09	0.026
Parental strain T1	− 0.32	0.06	< 0.001	0.12	0.05	0.010
Positive rel. quality T1	0.01	0.02	0.412	–	–	–
Negative rel. quality T1	− 0.02	0.02	0.070	–	–	–
Parental strain T3	–	–	–	0.08	0.05	0.096
Positive rel. quality T3	–	–	–	− 0.02	0.02	0.181
Negative rel. quality T3	–	–	–	0.02	0.02	0.216
Child emotional well-being						
Child emotional w.-b. T1	− 0.67	0.07	< 0.001	0.21	0.04	< 0.001
Parental strain T1	0.32	0.05	< 0.001	− 0.17	0.03	< 0.001
Positive rel. quality T1	− 0.01	0.01	0.310	–	–	–
Negative rel. quality T1	− 0.02	0.01	0.055	–	–	–
Parental strain T3	–	–	–	− 0.07	0.03	0.031
Positive rel. quality T3	–	–	–	0.02	0.01	0.382
Negative rel. quality T3	–	–	–	− 0.05	0.01	0.049

#### Child problem behaviors

The model revealed an acceptable model fit with RMSEA = 0.08, SRMR = 0.16, CFI = 0.86. Child problem behavior and parental strain at T1 showed negative effects on the change in problem behavior from T1 to T2 (strong effect of child problem behavior and moderate effect of parental strain) and weak positive effects on the change in problem behavior from T3 to T4. That means, the greater child problem behavior and the greater parental strain at T1, the greater the decrease in child problem behavior from T1 to T2 and the greater the increase from T3 to T4.

#### Child emotional well-being

The model revealed an acceptable model fit with RMSEA = 0.07, SRMR = 0.09, and CFI = 0.85. Child emotional well-being at T1 negatively predicted the change from T1 to T2 (strong effect) and positively predicted the change from T3 to T4 (moderate effect). That is, the lower emotional well-being at T1, the greater the increase in emotional well-being from T1 to T2 and the greater the decrease from T3 to T4. Parental strain at T1 positively predicted the change from T1 to T2 (moderate effect) and negatively predicted the change from T3 to T4 (weak effect). That means, the greater parental strain at T1, the greater the increase in child emotional well-being from T1 to T2 and the greater the decrease from T3 to T4. Additionally, parental strain at T3 and negative relationship at T3 showed weak negative effects on the change from T3 to T4, meaning the greater parental strain and negative relationship quality at T3, the greater the decrease in child emotional well-being from T3 to T4.

### Exploratory analyses on family-related well-being

Descriptives of family-related well-being (Fig. 1) are suggestive for greater interindividual variance during periods of lockdowns (T1, T4) compared to periods of loosened restrictions (T2, T3). Some families even seem to have higher family-related well-being during lockdown (T4) compared to pre-lockdown (T3). This is an interesting pattern to follow up as it could reveal which families do particularly well during a lockdown. To this end, we computed exploratory analyses to examine characteristics of families who did better during lockdown compared to pre-lockdown periods. We split families in two groups: families who reported increased family-related well-being (difference score T4–T3 > 0, *n* = 174) were compared against families who reported equal or decreased family-related well-being (difference score T4–T3 <  = 0, *n* = 301). Independent sample *t* tests suggest that families differed with regard to age of the child reported on and number of children living in the household. Families who reported increased well-being during lockdown compared to pre-lockdown reported on average on younger children (*M* = 5.5, SD = 2.0), *t*(473) = 2.01, *p* = 0.045, and showed a tendency to having less children (*M* = 1.9, SD = 0.6),* t*(473) = 1.76, *p* = 0.079, compared to families who reported equal or decreased family-related well-being (age: *M* = 5.9, *SD* = 2.0; children in household: *M* = 2.0, *SD* = 0.7). For the first lockdown, the absolute value of family-related well-being is informative, because the variable reflects family-related well-being in comparison to before the pandemic, with scores > 4 indicating an improvement. Across all participants, 56% (991) families reported improved family-related well-being compared to before the onset of the pandemic. Divided by age, that was the case for 54% (655) of families with younger children (aged 3–6 years) and 59% (336) of families with older children (aged 7–10 years). These findings suggest that occasionally families also profitted from the lockdown period. Notably, this analysis was exploratory and needs further confirmatory investigation.

## Discussion

The current study investigated child psychological well-being and mental health during the COVID-19 pandemic across four measurement occasions relying on an ecological A (lockdown)–B (relaxation)–B (relaxation)–A (lockdown) design. This allowed us to differentiate long-term (i.e., of the pandemic generally independently of lockdowns) from short-term effects (i.e., specific effects of lockdowns) on trajectories of child well-being. Importantly, our results demonstrate that adjustment trajectories vary by domain. While child problem behavior and emotional well-being recovered during relaxation periods (wave-like trajectory), child family-related well-being steadily decreased over the course of the pandemic (linear trajectory). This suggests the presence of different change processes at the individual and social level. Parental stress emerged as risk factor amplifying negative effects of the pandemic on child psychological well-being. Importantly, low levels of negative aspects in the parent–child relationship quality constituted a protective factor buffering against pandemic-related effects. Thus, the present study moves the field forward by differentiating short-term from long-term effects of the pandemic and by uncovering risk and resilience factors for trajectories of child well-being across lockdowns and relaxations.

Notably, our results point to the coexistence of short-term effects that quickly change as the situation evolves (e.g., child emotional well-being and problem behavior) and long-term effects of the pandemic that span periods of lockdowns and relaxations alike (e.g., family-related well-being). The short-term effects underscore developmental systems theory [39] and could point to the importance of friendships and peer relationships for child socioemotional adjustment in times of crisis (e.g., [29, 38]) as peer interaction were considerably less restricted during relaxations. Contrastingly, the long-term effect suggests that during the first lockdown, family-related well-being was elevated and then steadily decreased across time. Reasons for improved family-related well-being particularly during the first lockdown might be the increased time parents and children spent at home due to the very strict curfew regulations and the fact that there was no distance learning yet to manage by caregivers and children during the first lockdown in Germany. However, this shared time might have decreased alongside with increasing pandemic-related frustration and insecurity as the pandemic dragged on, resulting in diminished levels of family-related well-being at later measurement points [7, 37]. At the same time, some small families with young children were able to profit from the second lockdown, possibly due to their increased resources for individual children and their lower reliance on educational institutions, especially schools.

Our findings extend previous longitudinal work in three ways. First, our findings expand longitudinal work showing that children not only adjusted positively in emotional and behavioral difficulties from lockdown to relaxation [6, 19, 31, 66], but that the reverse effect can be found from relaxation to lockdown. This relates well to other work reporting similar negative effects of decreasing psychological well-being and increasing internalizing and externalizing problems from pre-pandemic to pandemic [35, 77]. Second, our findings contribute to a growing body of literature suggesting decreasing family-related well-being [7] and increased prevalence in low quality of life from the first to the second lockdown [62], but see [63, 64] by showing that family-related well-being seems to follow a continually declining trajectory even spanning relaxation periods over the first year of the COVID-19 pandemic. Third, our work adds to research showing positive relations between pandemic-related stressors on later externalizing and internalizing symptoms [67] by indicating that parental strain constitutes a particularly substantial risk factor for children’s positive psychological adjustment to the pandemic. Thus, our study specifically complements previous leading-edge work on child and family mental health during the pandemic in Germany [62-65]. Very few studies allowed for a more exhaustive picture. For example, Houghton and colleagues (2022) found longitudinal decreases in mental health particularly for adolescents without neurodevelopmental disorders (see also [23, 28] for longitudinal work with youth). Yet, the study was restricted to adolescence, and our work goes beyond that by focusing on early and middle childhood. Thus, the present work is among the first to separate short-term from long-term effects of the pandemic on child psychological well-being by relying on an ecological design.

Further, our results indicate that parental stress longitudinally led to a greater improvement in child well-being from lockdown to relaxation and to a greater deterioration of child well-being going from relaxation to lockdown. This suggests that higher parental stress is associated with higher volatilities in child well-being trajectories and thus constitutes an important factor explaining interindividual differences in child coping with the COVID-19 pandemic. Specifically, parental stress seems to amplify the impact of the pandemic on child well-being. On the other hand, the parent–child relationship quality emerged as important resilience factor attenuating negative effects of the pandemic independent of parental stress. This resonates well with and extends previous longitudinal findings demonstrating the negative impact of parental stress and hostile parenting on child well-being during the pandemic [19, 35, 66] and notions of close relationships as resilience factors [13, 20, 32, 48, 58, 69]. Thus, parental stress and the parent–child relationship quality constitute key variables in explaining interindividual differences in child developmental trajectories.

### Limitations and conclusion

While the present work exhibits multiple strengths, there are some limitations and directions for future research to consider. First, the present study relied on parental reports as especially younger children (3–6 years old) are limited in their cognitive abilities to accurately report on their general well-being and problem behavior. This is in line with previous longitudinal work assessing the effect of the COVID-19 pandemic on younger children in large samples [6, 7, 66, 78]. As educational institutions have largely reopened, future work should also include teachers’ reports and assess behavioral indicators of child well-being in smaller samples requiring individual testing sessions. Second, cross-national samples are called for to get a more complete picture of the overall child psychological well-being trajectories during the COVID-19 pandemic. Third, the four measurement points did not only differ in terms of lockdown measures but also in terms of other factors (e.g., mortality rates) that could have contributed to the findings. Specifically, there could also be seasonality effect with our design. Yet, given that we did not find meaningful differences between the two relaxation periods (summer, fall), we assume that it is unlikely that they explain the main effects of the study. Fourth, future work should assess the individual lockdown situation of children in more detail to investigate if and to what extent these factors contribute to the trajectories found in the current study. For example, children experiencing very strict social distancing measures in their families and long closures of educational and recreational facilities might evidence different trajectories from children less affected by social distancing and closures of education and recreational facilities.

Future research with samples of more heterogenous socioeconomic statuses is needed to generalize the findings of the present study. While the amendments of the SDQ and KIDSCREEN scales were necessary adaptations to accurately study the specific COVID-19 situation, this also limits the possibilities to compare our findings with other studies that used standardized and validated versions of the respective measures. While the current study sheds light on child emotional and family-related well-being during the COVID-19 pandemic, future work is needed to assess how pandemics affect child school-related and peer-related well-being and should also use children/youth report as a measure. Taken together, the present findings do not necessarily point to causal effects as the ecological design of the current study is unable to differentiate independent variables and confounding factors and the modification of some scales limits the psychometric quality of the results. Finally, our findings relate well to other longitudinal work not relying on a comparison in the item answering format, but on different methodological approaches (e.g., [6, 7, 19, 27, 62–64, 66, 77]). This gives us reason to assume the validity of our measures.

In conclusion, the current study is among the first to offer crucial evidence on the specific effects of lockdown measures on child psychological well-being and mental health during the COVID-19 pandemic in an ecological design. Taken together, the present work constitutes an important advancement of our efforts to gain a more complete picture of the effects of the COVID-19 pandemic on trajectories of child well-being.

### Supplementary Information

Below is the link to the electronic supplementary material.

Supplementary file1 (DOCX 33 KB)

## Data Availability

Data supporting the findings of this study are available under https://osf.io/5zrm2/
