# Supplementary material for: Short-term and long-term effects of the COVID-19 pandemic on child psychological well-being: a four-wave longitudinal study
Source: Eur Child Adolesc Psychiatry. 2023 Apr 29;33(3):909–22. doi: 10.1007/s00787-023-02215-7 (PMC10148581; doi:10.1007/s00787-023-02215-7)
Supplement: Supplementary file 1 — Supplementary file1 (DOCX 33 KB) [file 787_2023_2215_MOESM1_ESM.docx]

**Supplemental Material**

1. **Correlations of the Key Variables**

*Table 1:*

Zero-order correlations of key variables.

| Variable | 1 | 2 | 3 | 4 | 5 | 6 | 7 | 8 | 9 | 10 | 11 | 12 | 13 | 14 | 15 | 16 |
| --- | --- | --- | --- | --- | --- | --- | --- | --- | --- | --- | --- | --- | --- | --- | --- | --- |
| 1. T1 age child |  |  |  |  |  |  |  |  |  |  |  |  |  |  |  |  |
| 2. T1 parental strain | -.05* |  |  |  |  |  |  |  |  |  |  |  |  |  |  |  |
| 3. T1 emotional wb. | -.06** | -.51*** |  |  |  |  |  |  |  |  |  |  |  |  |  |  |
| 4. T1 family-related wb. | .03 | -.41*** | .49*** |  |  |  |  |  |  |  |  |  |  |  |  |  |
| 5. T1 problem behavior | -.08** | .49*** | -.58*** | -.38*** |  |  |  |  |  |  |  |  |  |  |  |  |
| 6. T2 parental strain | .18*** | .39*** | -.31*** | -.21*** | .33*** |  |  |  |  |  |  |  |  |  |  |  |
| 7. T2 emotional wb. | -.14*** | -.07* | .14*** | .12*** | -.09* | -.23*** |  |  |  |  |  |  |  |  |  |  |
| 8. T2 family-related wb. | .00 | -.08* | .12*** | .22*** | -.08* | -.21*** | .38*** |  |  |  |  |  |  |  |  |  |
| 9. T2 problem behavior | -.06 | .30*** | -.33*** | -.20*** | .67*** | .37*** | -.31*** | -.22*** |  |  |  |  |  |  |  |  |
| 10. T3 parental strain | .04 | .38*** | -.26*** | -.13*** | .25*** | .45*** | -.09 | -.13** | .26*** |  |  |  |  |  |  |  |
| 11. T3 emotional wb. | -.13*** | -.19*** | .23*** | .14*** | -.15*** | -.19*** | .26*** | .09 | -.17*** | -.37*** |  |  |  |  |  |  |
| 12. T3 family-related wb. | .10** | -.17*** | .12** | .20*** | -.15*** | -.12** | .04 | .20*** | -.15*** | -.29*** | .30*** |  |  |  |  |  |
| 13. T3 problem behavior | -.04 | .27*** | -.29*** | -.15*** | .61*** | .30*** | -.16*** | -.11* | .72*** | .37*** | -.29*** | -.20*** |  |  |  |  |
| 14. T4 parental strain | .04 | .47*** | -.27*** | -.18*** | .30*** | .39*** | -.09* | -.14** | .25*** | .38*** | -.21*** | -.15** | .29*** |  |  |  |
| 15. T4 emotional wb. | -.17*** | -.31*** | .45*** | .22*** | -.34*** | -.22*** | .21*** | .11* | -.25*** | -.25*** | .30*** | .14** | -.31*** | -.43*** |  |  |
| 16. T4 family-related wb. | -.05 | -.28*** | .20*** | .30*** | -.19*** | -.21*** | .07 | .22*** | -.15** | -.24*** | .18*** | .40*** | -.18*** | -.36*** | .37*** |  |
| 17. T4 problem behavior | .01 | .34*** | -.38*** | -.16*** | .66*** | .30*** | -.10* | -.04 | .64*** | .31*** | -.20*** | -.13** | .71*** | .41*** | -.57*** | -.30*** |

*Note.* * indicates *p* < .05, ** indicates *p* < .01, *** indicates p < .001.

1. **Questionnaire With the Key Study Variables**

**1. Parental Strain**

The following questions assess how much strain you experience in the current situation. The questions refer to the time period since the beginning of the curfew restrictions (T1)/ The questions refer to the last three weeks (T2-T4)

|  | Do not agree at all | Rather not agree | Part, part | Rather agree | Agree completely |
| --- | --- | --- | --- | --- | --- |
| I feel more strained in the current situation than normally |  |  |  |  |  |
| The current situation is more challenging for me than normally |  |  |  |  |  |
| I feel more stressed out in the current situation than normally |  |  |  |  |  |

**2. Child Well-Being – KIDSCREEN**

We ask you to indicate for each statement, if your child’s behavior or emotions have changed in comparison to the time before the curfew restrictions. Compared to before the pandemic and the associated restrictions, … (T1)/We ask you to indicate for each statement, if your child’s behavior or emotions have changed in comparison to the time before the COVID-19 pandemic. Think about the last three weeks in answering the questions. Compared to before the COVID-19 pandemic, … (T2-T4)

|  | Clearly less | Less | A little bit less | No difference | A little bit more | More | Clearly more |
| --- | --- | --- | --- | --- | --- | --- | --- |
| … my child enjoyed life in the last weeks (T1)/in the last 3 weeks (T2-T4) |  |  |  |  |  |  |  |
| … my child was in a good mood in the last weeks (T1)/in the last 3 weeks (T2-T4) |  |  |  |  |  |  |  |
| … my child had fun in the last weeks (T1)/in the last 3 weeks (T2-T4) |  |  |  |  |  |  |  |
| … my child was sad in the last weeks (T1)/in the last 3 weeks (T2-T4) |  |  |  |  |  |  |  |
| … my child felt so bad that s/he did not want to do anything in the last weeks (T1)/in the last 3 weeks (T2-T4) |  |  |  |  |  |  |  |
| … my child was lonely in the last weeks (T1)/in the last 3 weeks (T2-T4) |  |  |  |  |  |  |  |
| … my child was content in the last weeks (T1)/in the last 3 weeks (T2-T4) |  |  |  |  |  |  |  |
| … my child had time for himself/herself in the last weeks (T1)/in the last 3 weeks (T2-T4) |  |  |  |  |  |  |  |
| … my child was able to do things s/he wanted to do in its free time in the last weeks (T1)/in the last 3 weeks (T2-T4) |  |  |  |  |  |  |  |
| … my child felt that its parents had time for it in the last weeks (T1)/in the last 3 weeks (T2-T4) |  |  |  |  |  |  |  |
| … my child felt fairly treated by its parents in the last weeks (T1)/in the last 3 weeks (T2-T4) |  |  |  |  |  |  |  |
| … my child has been able to talk to its parents when s/he wanted in the last weeks (T1)/in the last 3 weeks (T2-T4) |  |  |  |  |  |  |  |

**3. Child Problem Behavior**

The following questions assess the situation of your child more closely. In answering please refer to the behavior of your child in the last 3 weeks.

|  | Not true | Somewhat true | Certainly true |
| --- | --- | --- | --- |
| Often complains of headaches |  |  |  |
| Has many worries |  |  |  |
| Often unhappy |  |  |  |
| Nervous or clingy |  |  |  |
| Has many fears |  |  |  |
| Often has temper tantrums |  |  |  |
| Generally obedient |  |  |  |
| Often fights |  |  |  |
| Often lies or cheats |  |  |  |
| Steals from home |  |  |  |
| Restless, overactive |  |  |  |
| Constantly fidgeting |  |  |  |
| Easily distracted |  |  |  |
| Reflects |  |  |  |
| Sees tasks through to the end |  |  |  |

**4. Parent-Child Relationship Quality**

How often do the following situations occur in general, independently of the current situation?

|  | Never | Seldom | Sometimes | Often | Very Often |
| --- | --- | --- | --- | --- | --- |
| My child tells me what s/he is thinking. |  |  |  |  |  |
| My child shares with me his/her thought and emotions. |  |  |  |  |  |
| The things my child does are getting acknowledged by me. |  |  |  |  |  |
| I show my child that I find it good. |  |  |  |  |  |
| I and my child are angry or furious towards each other. |  |  |  |  |  |
| My child and I are of different opinion and fight. |  |  |  |  |  |
| My child gets me to follow his/her lead |  |  |  |  |  |
| My child enforces its opinion when we cannot agree on something |  |  |  |  |  |
